# Over-Expression of the LH Receptor Increases Distant Metastases in an Endometrial Cancer Mouse Model

**DOI:** 10.3389/fonc.2013.00285

**Published:** 2013-11-19

**Authors:** Serena Pillozzi, Angelo Fortunato, Emanuele De Lorenzo, Elena Borrani, Massimo Giachi, Gianfranco Scarselli, Annarosa Arcangeli, Ivo Noci

**Affiliations:** ^1^Department of Experimental and Clinical Medicine, University of Firenze, Firenze, Italy; ^2^Istituto Toscano Tumori, Firenze, Italy; ^3^Department of Biomedical, Experimental and Clinical Sciences, University of Firenze, Firenze, Italy

**Keywords:** endometrial carcinoma, LH-R, LH, invasion, metastasis, mouse model

## Abstract

**Objective**: The aim of the present study was to define the role of luteinizing hormone receptor (LH-R) expression in endometrial cancer (EC), using preclinical mouse models, to further transfer these data to the clinical setting.

**Materials and Methods**: The role of LH-R over-expression was studied using EC cells (Hec1A, e.g., cells with low endogenous LH-R expression) transfected with the LH-R (Hec1A-LH-R). *In vitro* cell proliferation was measured through the WST-1 assay, whereas cell invasion was measured trough the matrigel assay. The effects of LH-R over-expression *in vivo* were analyzed in an appropriately developed preclinical mouse model of EC, which mimicked postmenopausal conditions. The model consisted in an orthotopic xenograft of Hec1A cells into immunodeficient mice treated daily with recombinant LH, to assure high levels of LH.

**Results**: *In vitro* data indicated that LH-R over-expression increased Hec1A invasiveness. *In vivo* results showed that tumors arising from Hec1A-LH-R cells injection displayed a higher local invasion and a higher number of distant metastases, mainly in the lung, compared to tumors obtained from the injection of Hec1A cells. LH withdrawal strongly inhibited local and distant metastatic spread of tumors, especially those arising from Hec1A-LH-R cells.

**Conclusion**: The over-expression of the LH-R increases the ability of EC cells to undergo local invasion and metastatic spread. This occurs in the presence of high LH serum concentrations.

## Introduction

Endometrial carcinoma (EC) is nowadays the most common gynecologic malignancy and the most frequent among infiltrating tumor of the female genital tract (1). Although EC is generally considered not aggressive, with a good prognosis and an overall survival (OS) rate of about 80%, there is still a group of patients with a high risk of recurrence and decreased OS (1–3). The latter is mainly related to the extra-pelvic spread of the cancer, with the onset of distant metastases, mainly to the lung (4, 5). Hence, further advances in controlling local and distant metastatic disease of EC are needed. As an example, it is worth noting that the methylation profile of EC can help to distinguish malignant from benign lesions (6).

It is known that ECs arising in the menopause are more aggressive and usually unlinked to estrogen secretion. It has been previously reported that estrogen independent ECs might be sensitive to elevated levels of luteinizing hormone (LH) (7), an event that characterizes the postmenopause. This hypothesis was first supported by the demonstration by Lin et al. that the gene encoding the LH receptor (LH-R) was expressed in primary human ECs (7, 8) and that the addition of LH influences proliferation of EC cell lines (9, 10). Afterward, our group better defined the role of LH in tumor progression of EC, demonstrating that LH is able to induce an *in vitro* invasive phenotype to EC cells (11). Such effect relies on the activation of LH-R and hence of protein kinase A (PKA), which in turn induces a functional activation of beta 1 integrin receptors and the subsequent secretion of active matrix metalloproteinase-2 ending in the triggering of cell invasiveness (11).

We also provided evidence that LH-R mRNA is expressed in the great majority of a cohort of primary ECs and that cells obtained from primary ECs can be triggered to invade by LH addition (12). A good correlation was found between the level of LH-R mRNA expressed by primary EC and the degree of LH-induced cell invasiveness *in vitro* (12, 13).

However, the role of LH-R in *in vivo* models is currently lacking.

The aim of the present study was to define the role of LH-R expression in EC, using preclinical mouse models, to further transfer these data to the clinical setting.

## Materials and Methods

### Cell culture and transfection

The human cell line Hec1A were grown in DMEM High Glucose (DMEM) (EuroClone) supplemented with 2% l-Glut, 10% fetal bovine serum (FBS) (Hyclone) and 1% penicillin/streptomycin. To establish transfected cell lines, the rLH-pcDNA III expression vector (a gift from Dr. Rodiem Patrice, Endocrinologie CHU d’Angers) and the empty expression vector (pcDNA III) were transfected into Hec1A cells with Lipofectamine 2000 (Invitrogen) according to the manufacturer’s instruction (Hec1A and Hec1A-LH-R). Forty-eight hours after transfection, cells were plated, and selected with G418 (800 μg/ml) for 2 weeks. The medium was changed every 3 days. Clones were expanded for further analysis.

### RNA isolation, cDNA synthesis, and real-time quantitative PCR

Total RNA, cDNA synthesis, and real-time quantitative PCR (RQ-PCR) for the quantification of LH-R mRNA were performed as reported in Ref. (12).

### Proliferation assay

Cell proliferation was evaluated by a colorimetric assay WST-1 (Roche). For this procedure, cells were synchronized by overnight serum starvation and seeded in 96-well plates at a density of 2.5 × 10^4^ cells/100 μl, in DMEM + 2%l-Glut + 10% FBS. Cells were incubated for 24 h in the presence/absence of human recombinant LH (rLH) (Ovitrelle^®^) at the concentration of 0.3 UI/ml. Assays were performed by adding WST-1 directly to the culture wells and incubating the plates for 20 min at 37°C. Plates were then read by ELISA microplate (EL_X_800 Universal microplate reader, Bio-TecK instruments) at 450 nm. All data were normalized to the absorbance value of the culture medium (DMEM + 2%l-Glut + 10%FBS). Experiments were performed in triplicate.

### Matrigel invasion assay

Hec1A and Hec1A-LH-R were resuspended in DMEM + 2%l-Glut containing 250 μg/ml heat-inactivated BSA (DMEM + BSA). Cell suspensions (200 μl) containing 50,000 cells, were seeded into the upper well of a Boyden chamber (NeuroProbe), separated from the lower compartment (filled with DMEM + BSA) by a porous membrane (8 μm pore diameter) previously coated with 50 μl of matrigel at the final concentration of 250 μg/ml (Becton Dickinson). Cells were incubated for 24 h in presence/absence of human rLH 0.3 UI/ml (Ovitrelle^®^). Boyden chambers were incubated at 37°C in humidified atmosphere containing 5% CO_2_ in air for 24 h. Cells remaining attached to the upper surface of the filters were carefully removed with cotton swabs. Migrated cells, which remained layered onto the lower face of the porous membrane, were fixed with absolute methanol at 4°C overnight and stained with Diff-Quick staining solution (Dade–Behring Holding GmbH). Cells were counted on the whole migration field, at ×40 magnification.

### *In vivo* models

All *in vivo* experiments were performed at the Laboratory of Genetic Engineering for the Production of Animal Models (LIGeMA) at the Animal Facility of the University of Firenze.

#### Xenograft model

About 5 × 10^6^ cells (Hec1A and Hec1A-LH-R cell lines), resuspended in 100 μl culture medium were injected s.c. into the flanks of nude mice (females, 4 weeks old). Mice were divided into two groups for each cell line:

- group 1 (five mice/group) received saline;
- group 2 (five mice/group) received i.p. injections of rLH (Ovitrelle^®^) (5 UI/mouse) daily for 4 weeks. After 4 weeks of treatment mice were sacrificed.

#### Orthotopic model

Subcutaneously, xenografts were established by the injection of 5 × 10^6^ cells as reported above. For orthotopic implantation, 27 nude mice were anesthetized with i.p. 2% Avertin, and the lower abdomen was swabbed with 70% alcohol. A longitudinal incision of the lower abdomen of about 15 mm in length was made (medial laparotomy). The retro peritoneum was then opened and the uterus visualized. The model consisted in the implantation of one tissue block (1 mm^3^ and weighting about 40 mg each) onto the posterior face of the uterus making a pocket and fixed with a surgical suture. The choice to orthotopically implant a tissue block instead of cell suspension is to maintain the full metastatic potential of the original tumor (maintaining a 3D tissue architecture, cell cell contact and tumor angiogenesis) (14). The tissue blocks were derived from masses developed in nude mice injected s.c with Hec1A or Hec1A-LH-R cells. The organs were reintroduced into the abdominal cavity and the retro peritoneum and skin were then closed with surgical suture. Mice for each cell line were divided in two groups and treated with 5 UI/daily of r-LH (Ovitrelle^®^) or saline solution in the case of control group. Seven weeks later, the animals were sacrificed by cervical dislocation for necropsy. Then, for all of the animals, the peritoneal cavity was opened and examined macroscopically.

### Histology and immunostaining

Samples of different organs were fixed in formalin and processed for routine histological examination. Histological analysis was carried out on 5 μm paraffin sections stained with hematoxylin and eosin (H&E). For immunohistochemistry (IHC), slides were stained with the following antibody: anti-MHC class I antibodies (H-300) (IHC: 1: 100) (Santa Cruz) [as previously described in (15)].

### Quantitative determination of LH concentration in nude mice

Mice (three mice per group) were treated with rLH (Ovitrelle^®^) at the dose of 5 UI, daily, or with saline. LH serum levels were measured 6 h after i.p. injection of the drug, collecting blood samples (100 μl) and quantifying LH concentrations with the LH Enzyme Immunoassay Kit (Genway, Biotech Inc), following manufacturer’s instruction. High and stable LH serum concentrations were detected in the rLH–treated vs. the control group: 10.94 ± 2.31 vs. 0.02 ± 0.01 ng/ml; *p* < 0.01 (Student’s *t* test).

### Statistical analysis

All averaged data are presented as mean ± SEM. The statistical significance of differences between experimental group was calculated with the Mann–Whitney test, Kruskal–Wallis Test, or Student’s *t* test, with a *p* < 0.05 being considered as statistically significant.

## Results

**Figure 1:**
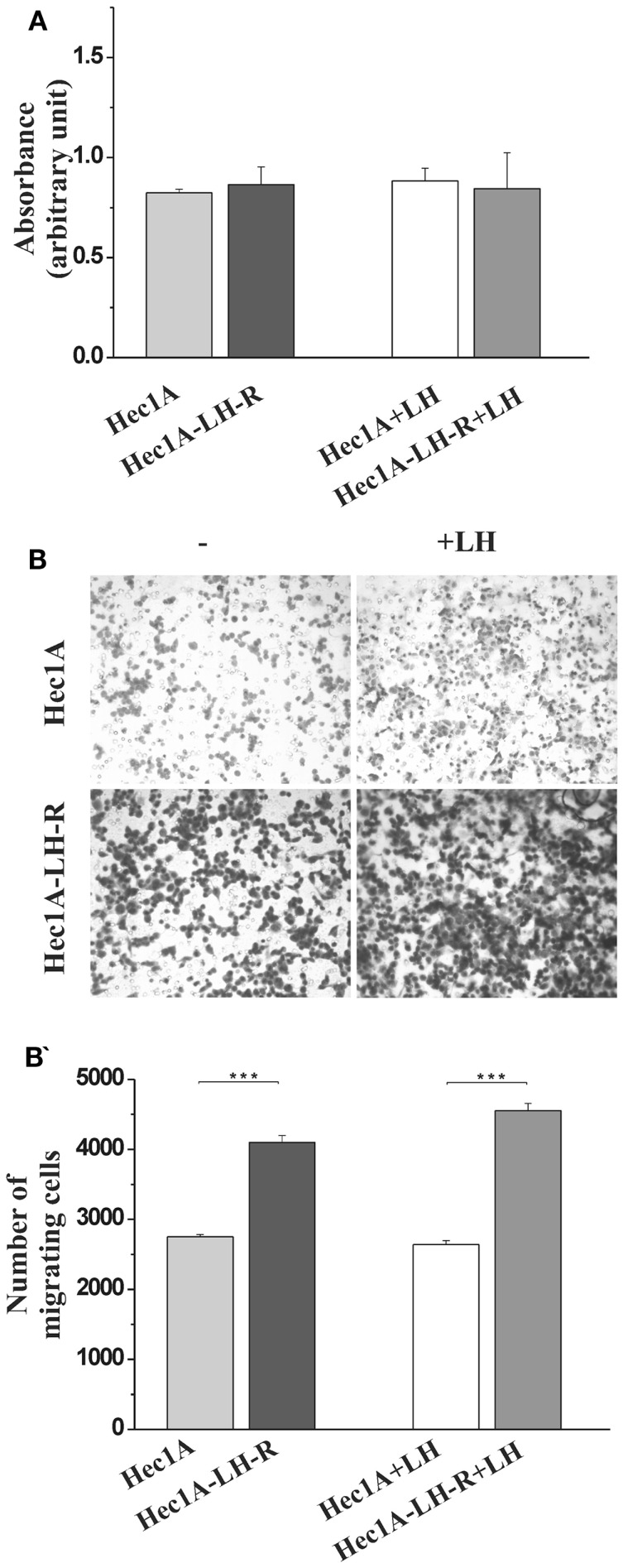
**Characterization of cell line over-expressing LH-R *in vitro* and *in vivo*: cell proliferation and invasion**. **(A)** Cell proliferation of Hec1A and Hec1A-LH-R cells. Cells (2.5 × 10^4^ cells/100 μl) were cultured in 96-well plates. After 24 h incubation, cell proliferation was evaluated with the colorimetric WST-1 assay (the absorbance value is reported on *y* axes). Histograms represent the mean of the absorbance value ± SEM (error bars). Statistical analysis was performed with Student’s *t* test. **(B,B′)** Cell invasion of Hec1A and Hec1A-LH-R cells. Cell invasion was evaluated using a Boyden chamber system where a Matrigel-coated porous membrane (8 μm pore diameter) was inserted as described in Section “Materials and Methods.” At time 0, recombinant LH was added or not to the medium. The number of migrated cells was determined after 24 h of incubation. Cells were fixed and stained with Diff–Quick staining solution, and counted on the whole migration field at ×40 magnification. In **(B)** pictures relative to representative membrane for Hec1A and Hec1A-LH-R cells, in the presence or in the absence of LH, are reported. Histograms in **(B′)** represent the mean of migrated cells ± SEM (error bars). Statistical analysis was performed with Student’s *t* test.

We produced Hec1A cells, e.g., an EC cell line with low endogenous expression of LH-R (11), transfected with the human LH-R cDNA in the pcDNA III expression vector. The expression of the LH-R transcript in the transfected cell lines, quantified by RQ-PCR, was 13-fold higher than in Hec1A cells. Both cell proliferation and invasion were tested in either cell lines, in the absence or in the presence of rLH (0.3 UI/ml). The proliferation rate of Hec1A-LH-R cells was not significantly different from that of control Hec1A cells, and it was not affected by LH treatment (Figure 1A). On the contrary, the invasion capacity of Hec1A-LH-R cells was significantly higher than control Hec1A cells, either in the absence or in the presence of external LH (Figures 1B,B′).

These data confirmed previous indications (11, 12) that LH-R over-expression affects EC cell invasiveness, more than proliferation.

We then developed a mouse preclinical model of EC which also mimicked the hormonal conditions of the menopause (i.e., high levels of circulating LH) which is the period of higher incidence of EC. The model consisted in the implantation of a tissue block (1 mm^3^), derived from subcutaneous masses developed in nude mice injected with EC cells. Either Hec1A cells or Hec1A-LH-R cells were nude to develop subcutaneous masses into the posterior face of the uterus of nude female mice. Nude female mice show severe deficiencies in the reproductive functions such as delayed ovulation and ensuing sterility, and have undetectable concentrations of LH in the serum as previously reported (16) and confirmed in our model by LH Enzyme Immunoassay Kit determination (see Materials and Methods for details). Hence, to obtain high and stable LH concentrations, mice were treated with rLH, daily, starting 3 days after the implantation of tumor blocks, for 7 weeks. The group of mice which did not receive LH treatment represented the “low LH group,” which mimicked the condition of “pharmacologically inhibited LH secretion.”

Hence we developed four groups of mice:

**Table:** 

		Cell line injected	Treatment with LH
Group 1	Hec1A control	Hec1A	+
Group 2	Hec1A low LH	Hec1A	−
Group 3	Hec1A-LH-R control	Hec1A-LH-R	+
Group 4	Hec1A-LH-R low LH	Hec1A-LH-R	−

**Figure 2:**
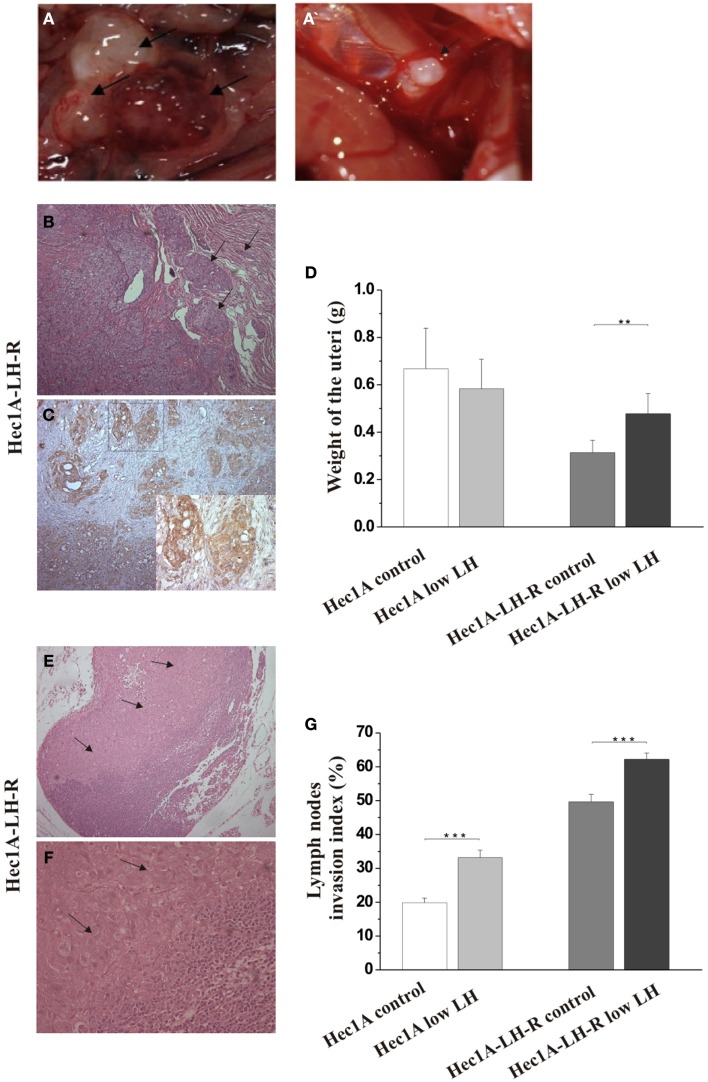
**Effects of LH-R over-expression and LH treatment in the orthotopic xenograft model of endometrial cancer (EC)**. **(A,A′)** Macroscopic view of representative examples of macrometastes detected at the para-aortic lymph nodes and at diaphragm respectively. **(B)** Histological characterization of the orthotopic EC model. Hematoxylin and eosin (H&E) sections showing orthotopic grafts of Hec1A and Hec1A-LH-R cell lines in direct contact and interaction with the uterus of the mice at the area of implantation and the myometrial infiltration by tumor cells (20×). **(C)** Immunohistochemistry staining of cancer cells in the myometrial wall. Staining was performed with anti-MHC class I antibodies (H-300), 1: 100, Santa Cruz (20× magnification). The insert in **(C)** is a magnification of the area indicated by the square. Histograms in **(D)** represent the mean weight of the uteri (±SEM) of mice implanted with Hec1A-LH-R cells or Hec1A and treated or not with LH. Statistical analysis was performed with Student’s *t* test. **(E)** Histological analysis showed para-aortic lymph node tumor cells invasion (see arrows) (10× magnification). **(F)** Magnification (40×) of the tumor invasion area (arrows). **(G)** Morphometric analysis of invasion of lymph nodes in between all the different treatments was performed and histograms reported represent the mean of the area of invasion/microscopic field (±SEM) of lymph nodes in between all the different treatments.

In any case, 4 weeks after the tumor implant, all the mice were euthanized and the peritoneal cavities were explored to evaluate the tumor engraftment into the uterus as well as the presence of macro metastases (Table 1). Representative examples of macro metastases detected are reported in panel 2A. The percentage of grafting of tissue blocks into the uteri was 93% (25/27). The uteri were surgically removed and weighted. Histological examination of the uteri showed myometrial infiltration in all the mice groups (Figure 2B). Immunohistochemical analysis using anti-human MHC antibodies further confirmed myometrial infiltration (Figure 2C). No difference in the extent of myometrial infiltration in the four groups was observed. The mean weight of the uteri of mice injected with Hec1A-LH-R and treated with LH (i.e., group 3) was lower than that of all the other experimental groups (Figure 2D). This fact can be traced back to a higher capacity of spreading out of, more than proliferating inside, the uterus conferred to EC cells by LH-R over-expression, and further potentiated by the high levels of serum LH obtained by treating the mice with rLH.

**Table 1:** **Evaluation of distant metastasis in the orthotopic experimental model of endometrial cancer**.

	Lymph nodes	Bladder	Spleen	Liver	Diaphragm	Lung
Hec1A control	5/5 (100)	3/5 (60)	1/5 (20)	0/5 (0)	1/5 (20)	1/4 (25)
Hec1A low LH	4/4 (100)	2/4 (50)	0/4 (0)	0/4 (0)	0/4 (0)	0/4 (0)
Hec1A-LH-R control	9/9 (100)	7/9 (78)	2/9 (22)	0/9 (0)	3/9 (33)	6/6 (100)
Hec1A-LH-R low LH	8/8 (100)	6/8 (75)	2/8 (25)	0/8 (0)	2/8 (25)	4/5 (80)

Screening for macrometastases in different organs of mice involved in the orthotopic experimental model of endometrial cancer. In the left column are reported the group of mice and different cell lines injected and treatment used. The number of mice with macrometastases and the total number of mice analyzed are separated by slashes. Percentages of mice with macrometastases are reported in parenthesis.

The statistical significance of differences between experimental group was calculated with Kruskal–Wallis test (non-parametric ANOVA).

Mice developed macro metastases in the para-aortic nodes in 50% of the cases. The histological analysis showed lymph node metastases in 100% of the cases (Table 1; Figure 2A). Lymph node lesions displayed histological and cytological features of the original tumor cells, including a solid growth pattern (Figures 2E,F). Lymph node invasion was quantified and the invasion index calculated: the invasion index of lymph nodes of mice implanted with Hec1A-LH-R in control (high LH) conditions was significantly higher than that of mice implanted with Hec1A in control conditions. No effect of LH withdrawal was observed (Figure 2G). Moreover, LH-R over-expression conferred a higher capacity of local invasion (i.e., bladder, spleen, and diaphragm) to EC cells (Table 1; Figure 2A).

**Figure 3:**
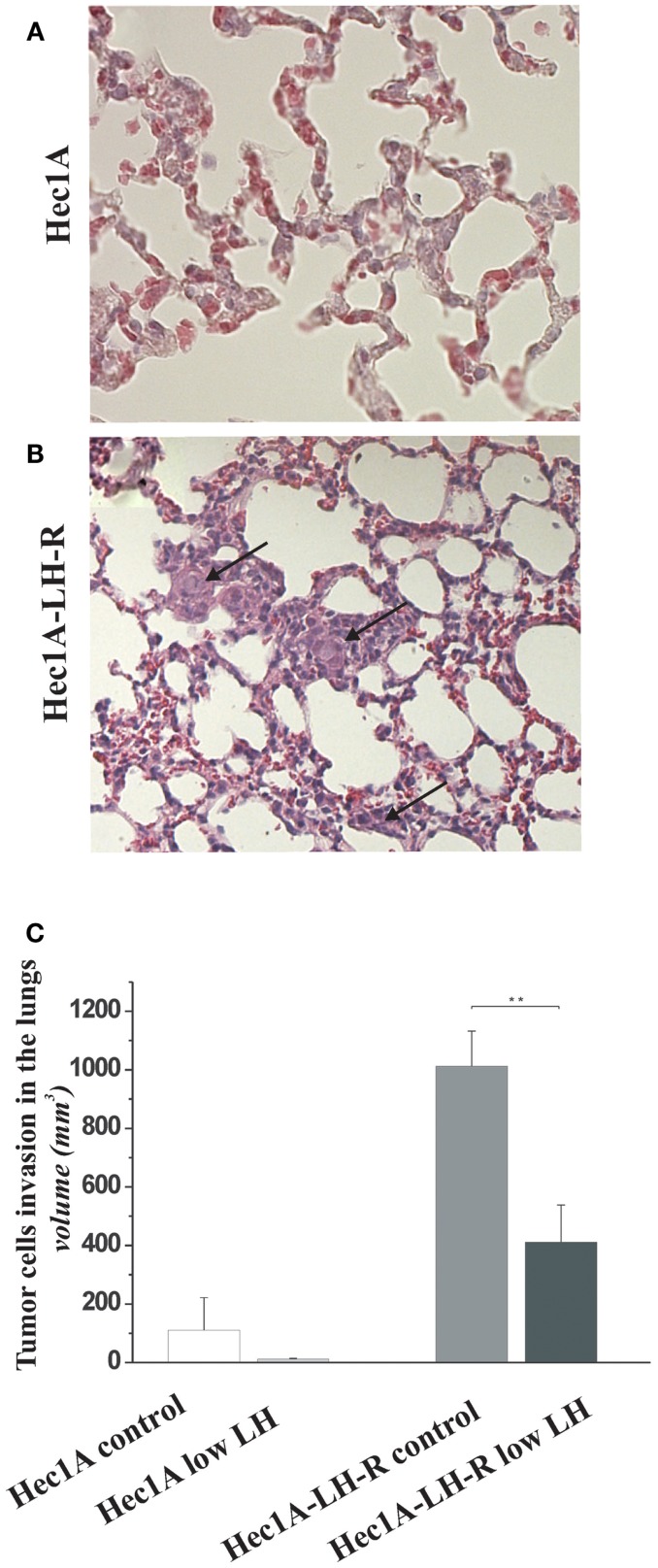
**Effects of LH-R over-expression and LH on distant metastases in the orthotopic xenograft model of endometrial cancer**. **(A,B)** Hematoxylin and eosin (H&E) sections of lung metastases from mice orthotopically engrafted Hec1A and from mice orthotopically engrafted Hec1A-LH-R. Tumor cells (see arrows) are present in the lung parenchyma and inside vessels (40× magnification). **(C)** Morphometric analysis of tumor cells invasion in the lungs. Histograms represent the mean of the area of invasion ± SEM (error bars). Mann–Whitney test has been performed (*p* = 0.0043). Asterisk indicate statistical significance;*p* < 0.05.

Finally, we evaluated the number of distant metastases in the lungs of the four groups of mice (Figure 3). The mice injected with Hec1A cells and treated daily with LH mimicking the condition of menopause, i.e., high levels of circulating LH (group 1) showed the capability, although at low levels, to develop distant metastases [25% of cases with lung metastases (Table 1)]. On the other hand, mice injected with Hec1A cells with undetectable levels of circulating LH (group 2), did not develop distant metastases (Table 1). The frequency of lung metastases increased significantly in mice injected with Hec1A-LH-R, 80% in mice with low levels of LH (group 4) and 100% in mice with high levels of circulating LH (group 3).

The morphometric analysis of the areas of tumor invasion allowed us to calculate the invasion index: the lungs of mice belonging to groups 1 and 2 showed a very low invasion index. Notably, mice injected with Hec 1A-LH-R (group 3) showed the highest pulmonary metastatization (100% of cases with lung metastases) and the highest lung invasion index (Figure 3A, see also arrows in Figure 3B). In these mice, LH withdrawal (group 4, i.e., Hec1a-LH-R low LH) produced a significant reduction in lung metastasization [both percentage of lung metastases and invasion index (see Figure 3C; Table 1)].

## Discussion

We evaluated the effect of LH-R on invasiveness of EC, using Hec1A cells, a human EC cell line which expresses the LH-R at low levels (11) as a model. In particular, we produced and characterized Hec1A cells stably transfected with, and hence over-expressing, the human LH-R (Hec1A-LH-R cells). In agreement with what previously observed (11), the proliferation rate *in vitro* of Hec1A-LH-R cells was not significantly different from that of Hec1A cells, either in the absence or in the presence of exogenous LH. On the other hand, cell invasiveness, triggered by LH addition, was significantly higher in Hec1A-LH-R cells compared to Hec1A cells. On the whole, these data confirm that a positive correlation exists between the level of LH-R expressed by EC cells (11) and primary EC samples (12) and cell invasiveness *in vitro*.

To better demonstrate this, and to have a model closer to what happens in the human setting, we developed an appropriate mouse preclinical model. The model consists in athymic nu/nu mice which received an implant of very small tumor block (1 mm^3^ and weighting about 40 mg each) in the uterine wall. The tumor blocks we implanted were obtained from subcutaneous masses obtained after injection of either Hec1A-LH-R or Hec1A cells. Athymic nude mice are a very good model for our goal, since they display severe deficiencies in reproductive function such as delayed ovulation and the females are usually sterile. Moreover gonadotropins are totally absent in these mice. Hence to make the model closer to the condition of menopause, i.e., high levels of circulating LH, the mice were treated daily with high doses of rLH. On the whole, the preclinical model we developed, not only provides the proper microenvironment, i.e., the uterus, which allows the growth of EC cells, as well as the emergence of subpopulations of tumor cells with higher invasiveness and metastatic potential, but also better mimicks those ECs which develop during the post menopause, when high levels of LH occurs.

The use of such model allowed us to conclude that (a) the over-expression of the LH-R on the membrane of EC cells, is critical to make EC cells to become more aggressive, and capable of invading the myometrium as well as surrounding organs, in particular para-aortic lymph nodes; (b) the concomitant over-expression of the LH-R and high, stable, levels of serum LH, make EC cells capable of reaching the lung and give rise to lung metastases; (c) the withdrawal of LH impedes EC cells to metastasize to the lung.

## Concluding Remarks

On the whole, the present paper identifies LH-R as a pro-metastatic molecular device in EC, further stressing previous suggestions (11, 12, 17). Moreover, data obtained in the orthotopic/menopausal mouse model strongly suggest the use of LH-R detection as a diagnostic tool to identify high risk EC patients. Finally, the possibility of treating LH-R positive, high risk EC cases, with Gn-RH analogs could be proposed.

## Authors Contribution

Serena Pillozzi and Emanuele De Lorenzo performed *in vivo* research, Angelo Fortunato, Elena Borrani, and Massimo Giachi perfomed *in vitro* assays, Annarosa Arcangeli and Ivo Noci designed the research, Annarosa Arcangeli and Serena Pillozzi wrote the manuscript, Gianfranco Scarselli edited the manuscript.

## Conflict of Interest Statement

The authors declare that the research was conducted in the absence of any commercial or financial relationships that could be construed as a potential conflict of interest.
